# Human & swine studies of concurrent 12-lead ECG & MRI

**DOI:** 10.1186/1532-429X-15-S1-P70

**Published:** 2013-01-30

**Authors:** Zion Tse, Charles Dumoulin, Ronald Watkins, Kim Butts Pauly, Israel Byrd, Jeffrey Schweitzer, Raymond Y Kwong, Gregory F Michaud, William Stevenson, Ferenc Jolesz, Ehud J Schmidt

**Affiliations:** 1 Engineering, The University of Georgia, Athens, GA, USA; 2 Radiology, Cincinnati Children's Hospital Medical Center, Cincinnati, OH, USA; 3 Radiology, Stanford University, Stanford, CA, USA; 4 Cardiovascular and Ablation Technologies, St Jude Medical Inc, St. Paul, MN, USA; 5 Cardiology, Brigham and Women's Hospital, Boston, MA, USA; 6 Radiology, Brigham and Women's Hospital, Boston, MA, USA

## Background

12-lead Electrocardiogram (ECG) is a clinical standard for patient physiological monitoring. An MRI-conditional 12-lead ECG should permit detection of acute myocardial ischemia during MR imaging or MRI-guided therapy, which may improve the handling of patients with ischemic histories. MRI visualization of ischemic episodes can also enhance the understanding of ischemic progression. Previously an MR-conditional 12-lead ECG system was presented. The system was equipped with Gradient-Ramp&RF (GR&RF) noise removal hardware & Magnetohydrodynamic (VMHD) voltage-removal software that improved ST segment visualization [1]. The study objectives were to (1) validate simultaneous 12-lead ECG monitoring & cardiac MR imaging in human subjects; (2) detect S-wave to T-wave (ST) ECG elevation & perform MR imaging of a Left Anterior Descending (LAD) balloon occlusion from the onset of ischemia to death in a swine model.

## Methods

**Figure 1:**
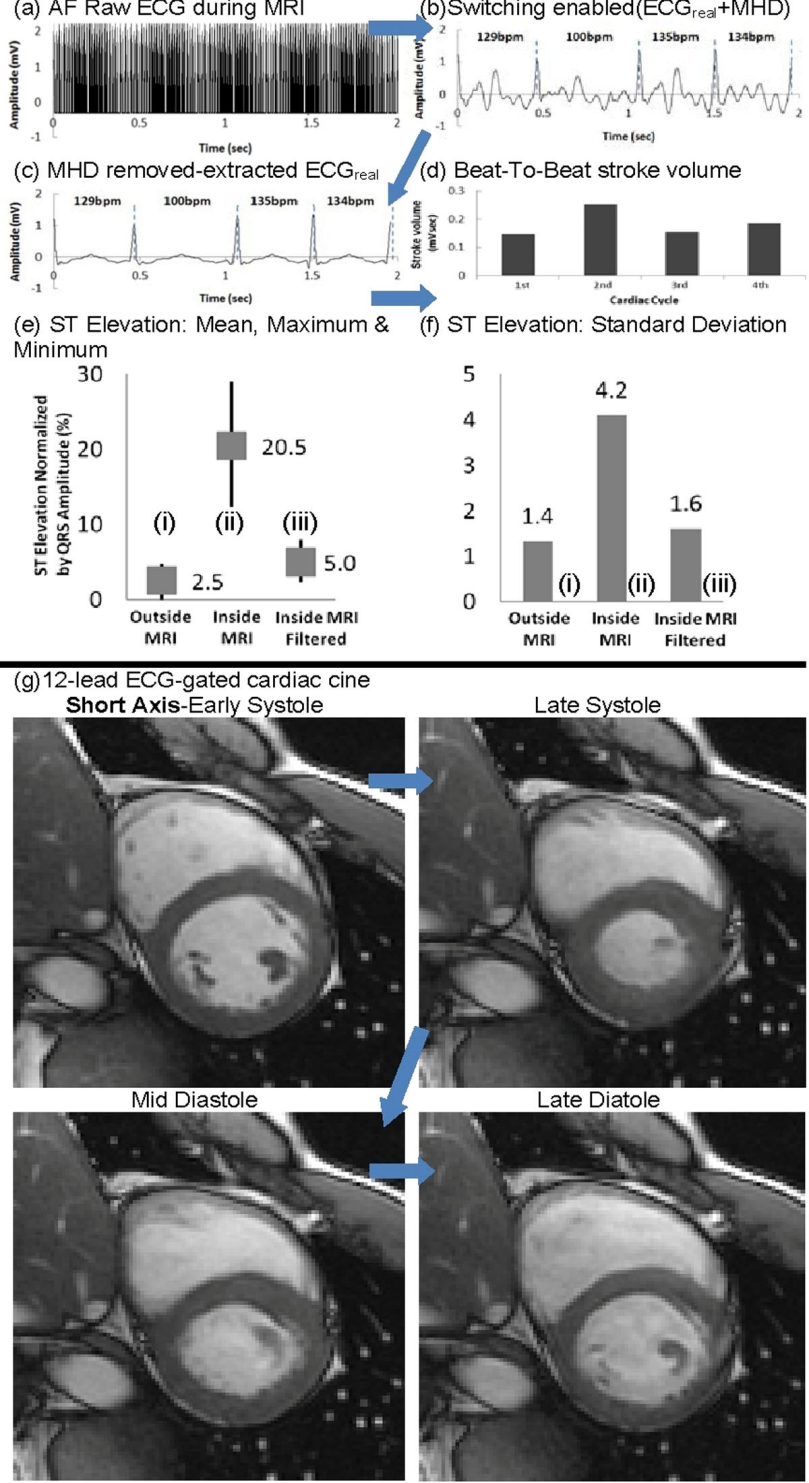
ECG processing of an AF patient in a 1.5T MR. In (b) MR imaging noise is removed. In (c) VMHD is removed & (d) Stroke volume is provided. (e) The ST elevation mean increased by only 2.5%-5% (0.062-0.13mV) between ECGs taken (i) outside MRI and (iii) inside MRI with ECG filtered. (f) The corresponding standard deviations are close (1.4-1.6), showing that the filtered ECG taken in (iii) is very close to the true ECG in (i). VMHD in (iii) was effectively removed, extracting real ECG. (g) 12-lead ECG-gated cardiac cine in a subject whose 4-lead ECG gating failed due to a strong MHD peak voltage which eclipsed the QRS complex.

**Figure 2:**
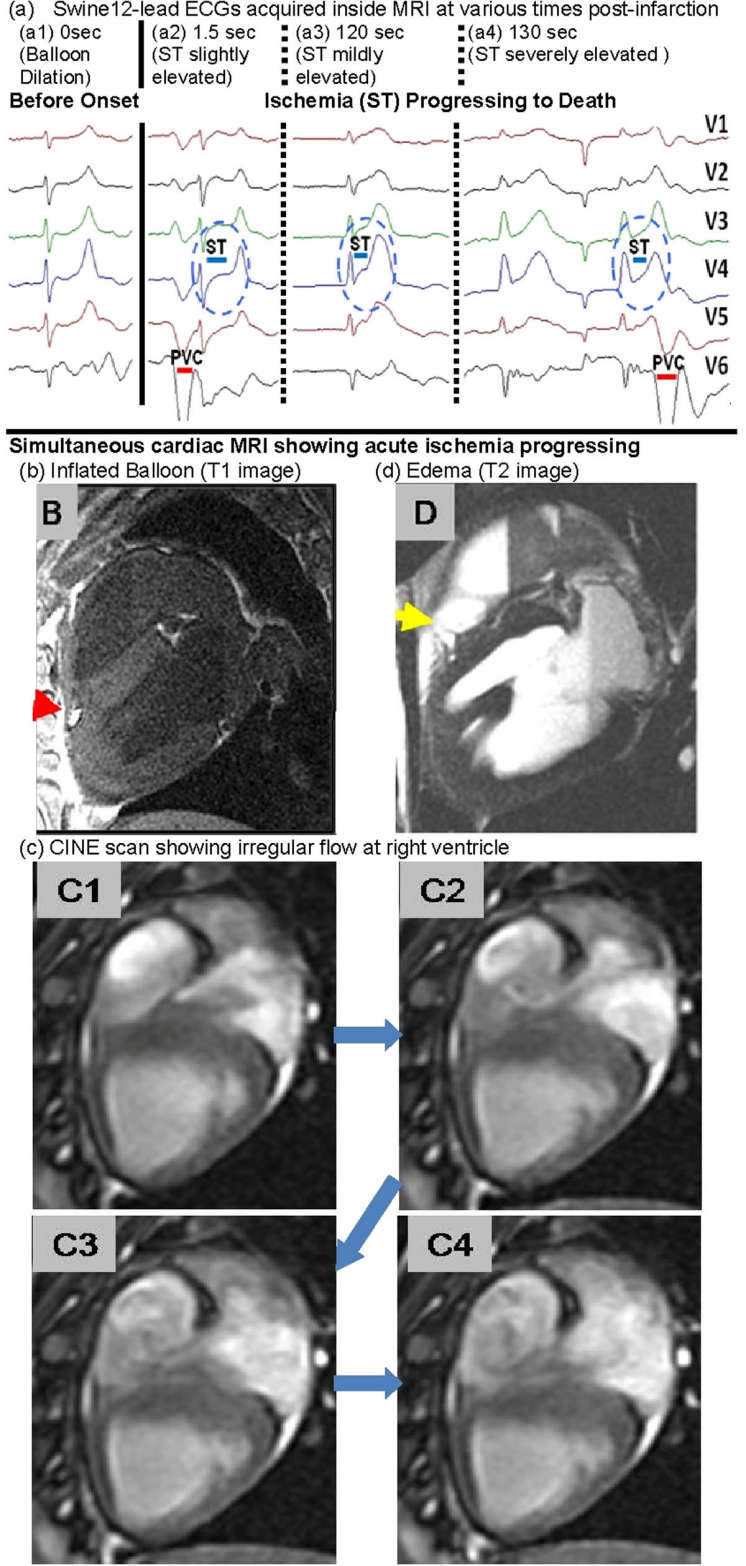
(a) Evolution of ST elevation over time, occasional PVCs and development of bradychardia are clear. (b) Inflated balloon position. (c1-4) Irregular flow patterns during ischemia at Δt=120-140sec. (d) Epicardial edema proximal to occlusion point.

1) Human Studies (Fig1) - 12-lead ECG & MRI in 14 subjects (including 2 premature ventricular contraction & 2 atrial fibrillation (AF) patients): Cardiac imaging was performed in a GE 1.5T MRI, with scans triggered by the 12-lead ECG & with simultaneous ECGs recording (1a-d). The derived real ECGs were compared to ECGs measured outside MRI for validation (1e-f).

2) Swine Study (Fig2) - Acute Ischemia Progressing to Death: A 2-mm balloon catheter filled with Gadolinium-doped water was inserted into the swine's distal LAD using X-ray guidance. The swine was moved to the MRI where continuous 12-lead ECG monitoring (2a) and cine imaging (2b-d) were performed. At t=0 seconds, the balloon was inflated to 20 atmospheres. MRI & simultaneous ECG monitoring were maintained until death ~20 minutes later. The balloon's position was confirmed using post-mortem 3D T1 imaging, & T2 imaging was used to detect edema.

## Results

1) AF patient's ECG processing & MRI (Fig1): (a) Raw ECG V6 was dominated by GR&RF noise during a GRE scan. (b) GR&RF noise was removed using the hardware circuit, leaving real ECG superimposed with VMHD. (c) Real ECG was extracted. (d) Beat-to-beat stroke volume (BTB-SV) was estimated from VMHD, where varying ventricular filling due to changing heart rates is responsible for irregular BTB-SV results. ST segment was well preserved for ischemia monitoring (c, e-f). The system outputted 100% accurate scan triggers at <30ms latency, allowing cine MRI in subjects where 4-lead ECG gating failed due to stronger VMHD peaks (g).

2) Acute Ischemia in Swine (Fig2): ST elevation was detected 1.5 seconds after onset (a1), progressing to acute ischemia (a2-3), bradycardia and death. Ventricular dysfunction and unusual flow vortexes were visualized with serial cine MRI (c1-4). Epicardial edema was observed adjacent to the balloon (d).

## Conclusions

MRI-conditional 12-lead ECG provides high-fidelity ECGs for robust cardiac-MRI. Acute ischemia detection is possible, with simultaneous MRI visualization of dysfunction progression.

## Funding

NIH U41-RR019703, R43 HL110427-01, AHA 10SDG261039
